# Normative Data for Blood Pressure in Croatian War Veterans: A Population-Based Study

**DOI:** 10.3390/ijerph18084175

**Published:** 2021-04-15

**Authors:** Mario Kasović, Zvonimir Kalčik, Lovro Štefan, Andro Štefan, Damir Knjaz, Marijana Braš

**Affiliations:** 1 Department of General and Applied Kinesiology, Faculty of Kinesiology, University of Zagreb, 10000 Zagreb, Croatia; mario.kasovic@kif.hr (M.K.); andro.stefan.dro@gmail.com (A.Š.); 2 Department of Sport Motorics and Methodology in Kinanthropometry, Faculty of Sports Studies, Masaryk University, 625 00 Brno, Czech Republic; 3 Home of War Veterans, Park ‘Stara Trešnjevka’, 10000 Zagreb, Croatia; zvonimirkalcik@gmail.com; 4 Department of Sports Kinesiology, Faculty of Kinesiology, University of Zagreb, 10000 Zagreb, Croatia; damir.knjaz@kif.hr; 5 Clinical Hospital Center ‘Zagreb’, 10000 Zagreb, Croatia; marijana.bras@mef.hr

**Keywords:** standards, hypertension, percentile, blood pressure, war veterans

## Abstract

*Purpose:* The current study aimed to investigate the normative data for blood pressure. *Materials and Methods:* From 2017 to 2020, 2032 men and women classified as ‘war veterans’ were recruited (mean age ± standard deviation (SD): 60.97 ± 7.98 years; mean stature: 172.50 ± 9.10 cm; mean body mass: 90.25 ± 36.45 kg; mean body-mass index: 29.66 ± 5.59 kg/m^2^; 29.9% women). Their systolic and diastolic blood pressures were measured three times. The procedure was carried out according to the American Heart Organization. The sex-specific and age-specific normative data for the 5th, 25th, 50th (median), 75th, and 90th percentiles for systolic blood pressure (SBP), diastolic blood pressure (DBP), pulse pressure (measured as SBP-DBP) and mid-BP (the average of SBP and DBP) were presented. *Results:* The men had higher SBP (*p* < 0.001), DBP (*p* < 0.001), pulse pressure (*p* < 0.001) and mid-BP (*p* < 0.001) compared to the women. The age-specific differences showed that older individuals had higher values of SBP (*p* < 0.001), pulse pressure (*p* < 0.001), and mid-BP (*p* < 0.001), while no significant differences for DBP (*p* = 0.496) were observed. *Conclusions:* This is the first study providing sex-specific and age-specific normative data for blood pressure in war veterans.

## 1. Introduction

Blood pressure is an essential component of everyday systematic examination in humans. In clinical practice, systolic blood pressure (SBP) and diastolic blood pressure (DBP) are the most commonly reported measures [1]. Observing them separately, studies have shown that high SBP and DBP are associated with increased cardiovascular risks [2] and premature mortality [3]. Furthermore, it has been well-documented that higher levels of BP are associated with reduced cognitive functioning, including alternations in cerebral perfusion and changing the ratio of the white matter [4], leading to structural brain changes, dementia disorders, and Alzheimer’s disease [5]. From SBP and DBP, an additional measure can be calculated, i.e., pulse pressure (SBP-DBP), which has been associated with a higher likelihood for developing cardiovascular diseases [1].

Previous evidence reported that the global mean age-standardized SBP and DBP in the general population were 127.0 mm/Hg and 78.7 mm/Hg in men and 122.3 mm/Hg and 76.7 mm/Hg in women, with higher SBP and DBP observed in Central and Eastern Europe [6]. Secular trends over the past 40 years have shown constant or slightly decreased values of SBP and DBP worldwide [6]. Of note, the American Heart Association has recognized five blood pressure ranges: (1) normal—less than 120/80 mm/Hg, (2) elevated—120 to 129 SBP and <80 mm/Hg DBP, (3) hypertension stage 1—130 to 139 SBP or 80 to 89 mm/Hg DBP, (4) hypertension stage 2—140/90 mm/Hg or higher, and (5) hypertension stage 3—180/120 mm/Hg or higher [7].

Hypertension, defined as SBP ≥ 140 mm/Hg or DBP ≥ 90 mm/Hg, has become a public health burden worldwide [8]. It 2010, the global age-standardized prevalence of hypertension was 31.1%, with slightly higher prevalence in men compared to women [9]. Hypertension has been consistently associated with cardiovascular diseases and mortality, independently of other risk factors [8].

Measuring SBP and DBP by sex and age is the first step of the correct classification and identification of individuals at higher risk. Therefore, reference-based standards for such measures are needed. To date, only a handful of studies have investigated reference data for BP [10,11,12,13], while little evidence has been provided for war veterans. It has been well-accepted that war can cause chronic stress and impaired health status [14]. A retrospective study on the prevalence of acute coronary syndrome before and during the 1992–1995 war in Bosnia and Herzegovina revealed that higher BP levels increased the frequency of acute myocardial infarctions and unstable angina pectoris cases during the war [15].

Therefore, the current study aimed to investigate the normative data for systolic and diastolic blood pressure in a large sample of Croatian war veterans.

## 2. Materials and Methods

### 2.1. Study Participants

In this cross-sectional study, we recruited men and women who participated in a homeland war between Croatia and Serbia from 1990 to 1995. All of the participants were part of the Home for Croatian Veterans. The Home for Croatian Veterans is an accommodation and rehabilitation institution established by the Ministry of Croatian Veterans with the intention of improving and strengthening the provision of comprehensive care for the veteran and suffering population. The general goal of the Home of Croatian Veterans is to preserve the *acquis* and mitigate the negative consequences of the Homeland War. The special goal refers to strengthening the care system and raising the quality of life of the veteran and suffering population through the activities of the Home of Croatian Veterans. The institution provides the user with the use of accommodation or accommodation services. The accommodation program includes the use of services during the provided accommodation in the Institution 24 h a day for up to 20 days. The program of stay implies the use of the services of the Institution during a full-day or half-day stay. A half-day stay can last up to 6 h a day. The full day stay can last from 6 to 10 h a day. The program of the stay does not include overnight stays in the Institution, and is intended for users who live near the Home and use the partial services of the Home. For the purpose of this study, we collected the data from 2017 to 2020 for all of the participants within the facility care. During the period of 4 years, approximately 2500 users used the accommodation service. Of these, 468 participants did not have their blood pressure measured and were excluded from further analysis. Our final sample consisted of 2032 facility users (29.9% women). Before the study began, all of the participants gave written informed consent for participation. All of the procedures were anonymous and in accordance with the Declaration of Helsinki, and were also approved by The Home for Croatian Veterans (Ethics code number: 2017/04).

### 2.2. Blood Pressure Measurement

Blood pressure was measured three times in a sitting position after a 5 min rest period using a standard mercury sphygmomanometer blood pressure cuff, according to the American Heart Association’s standardized protocol [1]. Specifically, blood pressure was measured three times by using a standard mercury sphygmomanometer on the right mid-arm at the same level as the heart. Previous evidence describes the protocol: “as the cuff is gradually deflated, blood flow is re-established and accompanied by sounds that can be heard with a stethoscope held over the brachial artery at the antecubital space” [1]. The average systolic and diastolic blood pressure was taken. The practitioner was not dressed as a doctor during the measurement, in order to simulate a home environment and discard the ‘white-coat hypertension syndrome’. Of note, no antihypertensive drugs were used prior to or during the study by the study participants.

### 2.3. Data Analysis

The basic descriptive statistics are presented as mean and standard deviation (SD). The Kolmogorov–Smirnov tests showed that the data were normally distributed. The sex and age differences were calculated by using analysis of variance (ANOVA) with a post hoc comparison test between the groups. For each variable, we determined sex-specific and age-specific percentile values (5th, 25th, 50th, 75th and 90th percentile) and used the Lambda (L), Mu (M) and Sigma (S) method, in which the optimal power to obtain normality is summarized by a smooth (L) curve, and trends in the mean (M) and coefficient of variation (S) are similarly smoothed. Next, all three curves (L, M and S) were summarized based on the power of age-specific Box–Cox power transformations for the normalization of the data. The LMS method assumes that the data can be normalized using a power transformation and by removing the skewness [16]. The sex and age differences were computed by using the analysis of variance (ANOVA) with post hoc comparisons and Bonferroni correction. All of the assumptions—including the level of Leven’s test of homogeneity, normal population distribution, and data independency—were met. All of the analyses were performed in Statistical Packages for Social Sciences version 23 (SPSS Inc., Chicago, IL, USA).

## 3. Results

The basic descriptive statistics are presented in Table 1. Men were taller and heavier compared to women. Interestingly, the body-mass index values showed no significant differences between the sexes. Heart, mental and metabolic diseases, and smoking and alcohol drinking were more prevalent in men, while a similar prevalence of respiratory diseases in both sexes was observed. The men showed significantly higher mean values in SBP, DBP, pulse pressure, and mid-BP compared to women (*p* < 0.001). Of note, no significant differences in blood pressure between the war veterans with and without diseases were observed (*p* = 0.174–0.486).

Table 2 shows the sex-specific and age-specific normative data for SBP, DBP and pulse pressure. The men had higher SBP ((*F* = 1, 2030) = 55.96, *p* < 0.001), DBP ((*F* = 1, 2030) = 35.41, *p* < 0.001) and pulse pressure ((*F* = 1, 2030) = 33.09, *p* < 0.001) compared to the women. The age-specific differences showed that older individuals had higher values of SBP ((*F* = 6, 2025) = 8.62, *p* < 0.001) and pulse pressure ((*F* = 6, 2025) = 15.98, *p* < 0.001), while no significant differences for DBP ((*F* = 6, 2025) = 0.90, *p* = 0.496) were observed. The sex and age interaction effect did not show significant differences in SBP ((*F* = 6, 2031) = 1.00, *p* = 0.424) and pulse pressure ((*F* = 6, 2031) = 0.46, *p* = 0.840), but a significant interaction in DBP ((*F* = 6, 2031) = 2.70, *p* = 0.013) was observed.

Table 3 shows the prevalence of ‘normal’, ‘elevated’, ‘stage 1 hypertension’, ‘stage 2 hypertension’ and ‘hypertensive crisis’ blood pressures. In both men and women, a significant correlation between age and blood pressure categories was observed (*r* = 0.08, *p* = 0.003 and *r* = 0.18, *p* < 0.001), pointing out that older participants suffered more frequently from higher blood pressure. The most prevalent categories were ‘stage 1 hypertension’ and ‘stage 2 hypertension’ in both sexes. Furthermore, when observing SBP as a numerical value, a significant correlation with age was found (*r* = 0.16, *p* < 0.001), while DBP was not significantly correlated with age (*r* = 0.01, *p* = 0.561).

## 4. Discussion

The current study aimed to investigate the normative data for systolic and diastolic blood pressure for Croatian war veterans. The main findings are: (1) the men had higher SBP, DBP and pulse pressure, compared to the women; (2) older individuals had higher values of SBP and pulse pressure, while no significant differences for DBP were observed; and (3) the highest prevalence of ‘stage 1 hypertension’ and ‘stage 2 hypertension’ in both sexes was noted.

This is the first study examining reference data for blood pressure parameters in war veterans. Indeed, cardiovascular diseases are the leading cause of mortality among male veterans in healthcare settings, and a higher prevalence of hypertension has been found in these individuals compared to general population [17]. The average values in SBP and DBP among the war veterans in our study are significantly higher compared to the previously published normative data for the general population [11,12,13]. Specifically, a study by Thijs et al. [11] showed that the average values for the SBP and DBP are 115/71 mm Hg in normotensive persons and 119/74 mm Hg in untreated subjects. Higher values of SBP and DBP in war veterans compared to general population can be explained by environmental factors, including job occupations and the health consequences of them [14]. Another potential factor remotely associated with BP is family member loss, given that it has been shown that arterial hypertension is more prevalent in those individuals with a killed relative compared to those without a killed relative [14]. The reason for the lack of normative data for the SBP and DBP in the population of war veterans is often explained by the unpredictability of the war and being unable to collect the data prior to combat situations [14].

Our results of the higher prevalence of hypertension are in line with previous studies [14,15,18,19,20]. For example, the prevalence of hypertension in 1996 and 2003 in the surviving family members during the war in Bosnia and Herzegovina was 53.1% (1996) and 50.7% (2003) with a relative killed, and 39.0% (equal for 1996 and 2003) without a relative killed [14]. Although the present study did not assess the number of relatives killed during the Homeland war, it can be postulated that more traumatic episodes may be associated with a higher incidence of hypertension. Interestingly, we found no significant differences in blood pressure between the participants with and without chronic diseases, which may lead to the conclusion that biological factors are not entirely responsible for higher blood pressure values, which are rather the fault of other environmental situations (the loss of a family member, stressful actions, etc.) in which the participants were engaged [12].

This study is not without limitations. First, blood pressure values should be obtained from longitudinal studies that give the possibility to assess natural changes in individual growth and development. Second, more detailed information about the environmental factors affecting blood pressure during the war was not collected, which might have led to different reference-based standards.

In conclusion, this is the first study aiming to develop the normative data for systolic and diastolic blood pressure in Croatian war veterans. The reported normative values serve different purposes. Participants in higher percentiles should be a target group for special interventions aiming to decrease the level of their blood pressure. Second, baseline results can be easily tracked by remembering a certain percentile at the beginning and after a follow-up period.

## Figures and Tables

**Table 1:** Basic descriptive statistics of the study participants (*N* = 2032).

Study Variables	Total (*N* = 2032)	Men (*N* = 1424)	Women (*N* = 608)	*p*-Value
	Mean ± SD	Mean ± SD	Mean ± SD	
Age (years)	60.97 ± 7.98	60.94 ± 7.77	61.04 ± 8.45	0.806
Stature (cm)	172.50 ± 9.10	177.05 ± 6.73	163.47 ± 5.89	<0.001
Body-mass (kg)	90.25 ± 36.45	96.24 ± 42.21	78.33 ± 14.70	<0.001
Body-mass index (kg/m^2^)	29.66 ± 5.59	29.81 ± 5.85	29.36 ± 5.04	0.303
Heart diseases (‘yes’ %)	47.00	49.30	41.60	0.002
Mental diseases (‘yes’ %)	29.90	36.90	13.30	<0.001
Metabolic diseases (‘yes’ %)	41.00	43.90	37.00	0.004
Respiratory diseases (‘yes’ %)	7.00	7.60	5.60	0.128
Smoking (‘yes’ %)	25.10	26.80	21.20	0.007
Alcohol drinking (‘yes’ %)	3.20	4.50	0.20	<0.001
SBP (mm/Hg)	134.80 ± 17.12	136.64 ± 17.14	130.49 ± 16.61	<0.001
DBP (mm/Hg)	83.69 ± 9.52	84.50 ± 9.57	81.78 ± 9.11	<0.001
Pulse pressure (mm/Hg)	51.11 ± 12.42	52.14 ± 12.73	48.70 ± 11.32	<0.001

**Table 2:** Sex-specific and age-specific normative data for blood pressure in the study participants (*N* = 2032).

Measure	Sex	Age	N	P5	P25	P50	P75	P90
SBP	Men	45–50	141	110	120	130	140	150
		51–55	216	110	120	130	140	160
		56–60	335	110	120	130	145	160
		61–65	349	120	130	140	150	160
		66–70	236	110	130	140	150	160
		71–75	94	110	130	140	150	160
		76–80	53	117	130	140	150	172
	Women	45–50	50	100	110	120	130	140
		51–55	88	104.5	120	130	130	140
		56–60	147	100	120	130	140	150
		61–65	155	110	120	130	140	150
		66–70	100	110	122.5	130	140	160
		71–75	38	109.5	130	140	150	160
		76–80	30	101	127.5	130	142.5	160
DBP	Men	45–50	141	70	80	85	90	98
		51–55	216	70	80	85	90	100
		56–60	335	70	80	85	90	95.2
		61–65	349	70	80	85	90	100
		66–70	236	70	80	85	90	100
		71–75	94	67	80	85	90	100
		76–80	53	70	80	85	90	100
	Women	45–50	50	60	80	80	80	90
		51–55	88	70	80	80	80	90
		56–60	147	60	80	80	90	90
		61–65	155	70	80	80	90	90
		66–70	100	70	80	80	90	99.5
		71–75	38	69.5	80	80	90	100
		76–80	30	65.5	80	80	90	90
Pulse pressure	Men	45–50	141	30	40	45	50	60
		51–55	216	40	40	45	60	60
		56–60	335	40	40	45	60	60
		61–65	349	40	50	50	60	70
		66–70	236	40	50	50	60	70
		71–75	94	40	50	60	60	70
		76–80	53	40	50	50	60	86
	Women	45–50	50	30	40	40	50	60
		51–55	88	30	40	50	50	60
		56–60	147	30	40	50	50	60
		61–65	155	38	40	50	50	60
		66–70	100	40	40	50	60	60
		71–75	38	30	40	50	60	70
		76–80	30	35.5	47.5	50	60	69

**Table 3:** The prevalence (%) of ‘normal’, ‘elevated’, ‘stage 1 hypertension’, ‘stage 2 hypertension’ and ‘stage 3 hypertension’ blood pressures in the study participants (*N* = 2032).

Sex	Age	*N*	Blood Pressure Categories				
			Normal	Elevated	Stage 1 Hypertension	Stage 2 Hypertension	Hypertensive Crisis
Men	45–50	141	4.30	2.80	47.50	44.00	1.40
	51–55	216	6.90	3.70	34.30	52.30	2.80
	56–60	335	4.50	2.10	37.00	54.30	2.10
	61–65	349	3.20	4.30	34.70	55.90	2.00
	66–70	236	4.70	2.50	26.30	61.00	5.50
	71–75	94	4.30	5.30	29.80	59.60	1.10
	76–80	53	1.90	3.30	37.70	47.20	9.40
Women	45–50	50	18.00	4.00	54.00	24.00	0.00
	51–55	88	9.10	6.80	53.40	28.40	2.30
	56–60	147	12.90	2.70	48.30	33.30	2.70
	61–65	155	12.30	3.90	42.60	39.40	1.90
	66–70	100	4.00	3.00	41.00	49.00	3.00
	71–75	38	2.60	0.00	28.90	68.40	0.00
	76–80	30	6.70	6.70	33.30	50.00	3.30

## Data Availability

All the data will be available from the corresponding author after reasonable request.
